# Importance-based approach to entrustable professional activities for psychiatric residency training

**DOI:** 10.1186/s12909-025-07655-0

**Published:** 2025-07-17

**Authors:** Wen-Yin Chen, Chih-Pang Chu, Tzu-Fu Huang, Hu-Ming Chang, Feng-Jung Kuo, Yu-Ting Liu, Sheng-Hsun Peng, Cheng-Jui Lee, Yan-Jiun Hung, Yueh-Pin Lin, Ming-Chyi Huang, Chian-Jue Kuo

**Affiliations:** 1 Department of Psychiatry, Taipei City Psychiatric Center, Taipei City Hospital, Songde branch, Taipei, Taiwan; 2 School of Medicine, College of Medicine, Fu Jen Catholic University, New Taipei, Taiwan; 3 Psychiatric Research Center, Taipei Medical University Hospital, Taipei, Taiwan; 4 Department of Health Promotion and Health Education, National Taiwan Normal University, Taipei, Taiwan; 5 Departmemt of Counseling Psychology, Chinese Culture University, Taipei City, Taiwan; 6 Department of Psychiatry, School of Medicine, College of Medicine, Taipei Medical University, Taipei, Taiwan; 7 Department of General Psychiatry, Taipei City Psychiatric Center, 309 Sung-Te Road, Taipei, 110 Taiwan

**Keywords:** Factor analysis, Competence-based medical education, Psychiatric residency training, Delphi method

## Abstract

**Background:**

Entrustable professional activities (EPAs) are essential tools in implementing CBME and integrating milestones into clinical practice. This study aimed to develop a set of EPAs tailored to the psychiatric residency training context with local institutional needs, and to dissect the differences and factor analysis in perceived EPA importance.

**Methods:**

21 customized EPAs specific to psychiatric residency training at Taipei City Psychiatric Center (TCPC) were developed based on the Taiwanese Society of Psychiatry (TSOP) framework. A two-round Delphi method was used to achieve consensus for the EPAs. The finalized EPAs were evaluated using an online questionnaire, and faculty members and residents rated each activity’s importance on a Likert scale of 0–10. Statistical analyses were performed on perceptions differences between teachers and trainees, and to cluster EPAs into meaningful competency clusters.

**Results:**

The finalized TCPC EPA framework included the TSOP’s original 17 EPAs and four additional EPAs to address advanced skills in psychotherapy, rehabilitation, legal applications, and innovative technologies. The total response rate was 89.6% for questionnaire assessing importance. Faculty consistently rated EPAs as more important than residents did, particularly clinical documentation, teaching and long-term care planning. Factor analysis revealed two loading factors, referring to internal knowledge and an external communication.

**Conclusion:**

The experience of adapting EPAs from global and national standards to fit the characteristics of a local training institution provides a valuable reference for the localization of EPA development. Observed differences in perceptions between trainees and teachers highlight the importance of aligning educational expectations. Enhancing awareness of the underlying dimensions associated with EPAs may help optimize psychiatric training outcomes for both trainees and instructors.

**Supplementary Information:**

The online version contains supplementary material available at 10.1186/s12909-025-07655-0.

## Introduction

Medical education has evolved to embrace competency-based medical education (CBME), which emphasizes students’ practical abilities and performance rather than focusing solely on the length of study or the number of completed courses [1]. The goal of CBME is to ensure that learners acquire the essential competencies needed to address real-world challenges in clinical practice. This approach helps translate the “core competencies” emphasized during training into the actual competence required in the workplace [2]. This shift is closely tied to the six core competencies for physicians established by the Accreditation Council for Graduate Medical Education (ACGME) in the United States. Using this core competency framework, curricula and assessments have been developed to strengthen these competencies [3, 4]. Milestones and entrustable professional activities (EPAs) are currently the most widely used assessment tools for implementing CBME in medical education and training [5]. Milestones focus on tracking the development of physicians’ competencies by defining stages of proficiency that residents must achieve in various clinical skills. EPAs, on the other hand, are units of professional work that trainees are expected to perform independently once sufficient competence is demonstrated. As EPAs are grounded in the concept of trust, assessment focuses on whether a supervisor feels confident to entrust the trainee with the task. Milestones and EPAs are complementary tools designed to integrate continuous assessment into clinical practice [6, 7].

CBME is currently being implemented in psychiatric residency training in Taiwan, drawing key references from the psychiatry milestones developed by the American ACGME workgroup [8] and the EPAs for psychiatry outlined by the Task Force of the American Association of Directors of Psychiatric Residency Training [9]. Since 2017, the Ministry of Health and Welfare has mandated that all specialty medical societies design CBME-based training and certification programs for specialists. In response, the Taiwanese Society of Psychiatry (TSOP) commissioned its education committee, chaired by Professor Cheng-Sheng Chen, to initiate the integration of CBME into the existing psychiatric specialist training and certification program. This initiative led to the collaborative development of the “Framework of EPAs and Milestone Development in Psychiatry Residency Training in Taiwan.” This framework, documented as the first draft by the TSOP, identifies 17 EPAs and has been published in an educational academic journal as a reference [10]. During its development, minor revisions and adjustments were made to the milestones to ensure alignment with Taiwan’s local healthcare context, enhancing both the relevance and practicality of the educational content. EPAs, in particular, are viewed as an opportunity to highlight the distinctive characteristics of each teaching hospital. The framework serves as a foundational blueprint for developing CBME programs tailored to the specific contexts of individual hospitals across Taiwan.

In Taiwan, qualified psychiatry training institutes include medical centers, regional teaching general hospitals, and psychiatric hospitals. Among these, approximately 54% of trainees are based in medical centers, 17% in regional teaching general hospitals, and 29% in psychiatric hospitals. According to a survey conducted as part of the TSOP CBME framework, agreement on several EPAs varied among trainees from these three types of training institutes [10]. As a result, the second step in developing CBME for psychiatry training in Taiwan involves engaging local hospitals in adapting the EPAs to reflect their unique educational characteristics. The Taipei City Psychiatric Center (TCPC), a division of Taipei City Hospital, specializes in psychiatric care, education, and research. Accredited in 1981 as Taiwan’s first psychiatric teaching hospital, TCPC offers comprehensive psychiatric services, including outpatient and inpatient care, community-based programs, and specialized clinics in addiction medicine, geriatric psychiatry, forensic psychiatry, and child and adolescent psychiatry. The initial version of the EPAs developed by TSOP was created by an expert panel based on general hospital settings. In response, TCPC established its own internal CBME development team in 2023 to create a localized version of the EPAs that better reflect the institution’s strengths. This effort also aligns with the second step of the TSOP CBME program, which encourages institutions to tailor EPAs to their specific teaching environments.

This study documents how the CBME team at a psychiatry-specialized teaching hospital identified and defined key EPAs, along with their perceived significance and importance. Analyses were conducted to explore differences in training emphases between psychiatric specialty hospitals and general hospitals for psychiatric residents, as well as discrepancies in the perceived importance of EPAs between trainees and supervisors. Additionally, factor analysis was employed to identify clusters of EPAs based on perceived importance, allowing the resulting dimensions to be introduced to both teachers and trainees. The findings may serve as a valuable reference for EPA development in other institutions. Furthermore, by documenting this process in the scientific literature, we aim to foster international dialogue and contribute to the advancement of CBME in psychiatry within Taiwan.

## Methods

### Data sources

The CBME development team at TCPC consisted of eight members: the director of the Psychiatry Residency Review Committee (RRC) training program, the director of medical education and research, the head of faculty development and a teaching-oriented attending physician, two core faculty attending psychiatrists, and three fifth-year psychiatry residents. The team undertook three main tasks: First, to review the 17 EPAs from the TSOP framework and evaluate their suitability for implementation at TCPC, including identifying any need for revision. Second, to examine international literature and discuss additional EPAs that could better reflect the unique characteristics of TCPC. Third, to assess the newly selected EPAs for clarity and relevance in their Chinese versions, followed by content validity evaluation. Finally, the perceived importance of all EPAs was evaluated using a questionnaire completed by all attending psychiatrists and psychiatry residents at TCPC.

The Delphi method is also known as the expert opinion method [11], was applied in two rounds for each of the aforementioned tasks. Detailed explanations of the objectives, topics, and requirements were provided to ensure that all experts fully understood the purpose and scope of each task. The TSOP draft EPAs were then subjected to a content validity review using the Content Validity Index (CVI) to evaluate their suitability. Feedback and suggestions were collected, quantified, and analyzed to reach consensus among members of the CBME team. A 5-point Likert scale was used to rate the suitability of each EPA, with higher scores indicating greater suitability. During each Delphi round, the percentage of mode responses and the quartile deviation (QD) were assessed. A suitability score below 4 or a mode percentage below 50% was considered to indicate a lack of consensus. EPAs with a CVI of ≥ 0.8 were considered to have reached acceptable content validity and were included in the finalized TCPC psychiatry EPA framework.

The TCPC psychiatry EPAs were then distributed through an online questionnaire to attending physicians and residents within the hospital. Respondents rated the importance of each EPA using a Likert scale (0–10, where 0 = strongly disagree, 5 = neutral, and 10 = strongly agree). At the time of the survey (January 2024), the hospital had a total of 48 physicians (29 attending psychiatrists, 16 resident physicians, and 3 research fellow psychiatrists). Of these, 43 completed the questionnaires, which corresponded to a response rate of 89.6%. The project was approved by the Research Ethics Committee of Taipei City Hospital (TCHIRB-11304019-E). Written informed consent was obtained from the TCPC CBME development team members. The need for consent to other participates was waived according to that (TCHIRB-11304019-E).

### Statistical analysis

A descriptive analysis was used to analyze the importance score between trainees (residents) and teachers (attending psychiatrists) in TCPC. Student’s *t*-tests were used to assess continuous variables. The normality of the data was assessed using the Kolmogorov–Smirnov test. For non-normally distributed data, we used a nonparametric Wilcoxon rank-sum test. We divided the EPAs as dichotomous items into those with full agreement (Likert scale = 10) and those without full agreement among the respondents regarding the importance and used a chi-squared test to analyze the differences between trainees and teachers. We corrected the variable of working experience (working years) with clinical practice by applying a regression model to examine the effect of being a trainee on the perception of importance among the EPAs. Finally, we used factor analysis to explore the underlying dimensions that explain the importance of the EPAs. All analyses were conducted using IBM SPSS version 24, and the significance level was set at *p* < 0.05.

## Results

### The process and outcome of local EPA development at TCPC

**Fig. 1:**
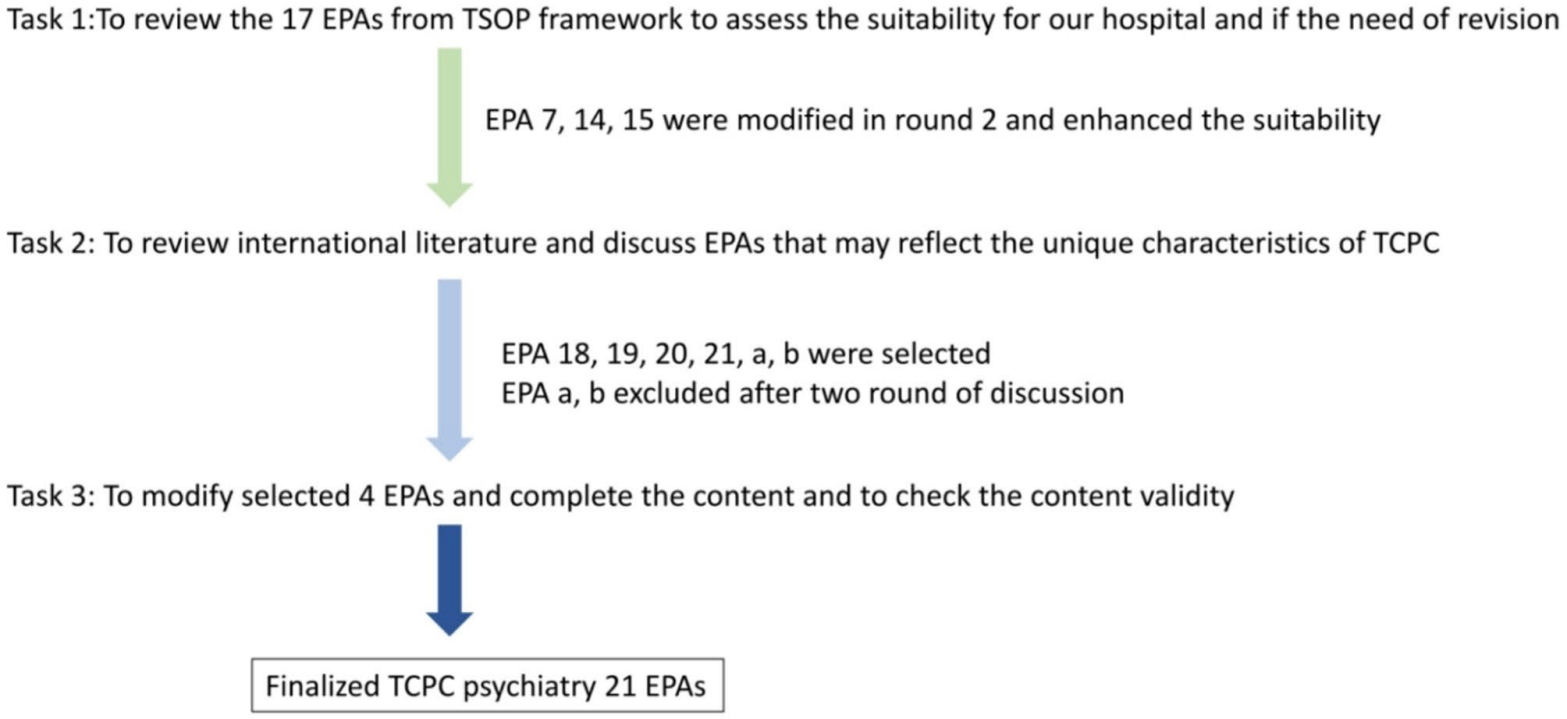
The process of psychiatric EPA development at TCPC with Delphi method

The process of developing psychiatry-specific EPAs at TCPC using the Delphi method is illustrated in Fig. 1. The mode percentages and QDs for ratings across the three tasks are presented in e-Table 1. In Task 1, all 17 EPAs originally derived from the TSOP framework were reviewed to assess their contextual suitability for implementation at TCPC. Fourteen of these EPAs achieved a CVI of 0.8 or higher in the first round of Delphi assessment, indicating acceptable content validity. The remaining three EPAs (EPA 7, EPA 14, and EPA 15) required revisions to their contextual descriptions and were re-evaluated in a second round, after which they successfully reached the consensus threshold. In Task 2 focused on the identification of additional EPAs that reflect the specific characteristics and training needs of TCPC. Six new EPAs were proposed, among which four—designated as EPAs 18 through 21—achieved strong content validity in the first Delphi round, with CVIs ranging from 0.875 to 1.0. Two other proposed EPAs—one concerning leadership in inter-professional healthcare teams and another related to the application of quality improvement methodologies in clinical practice—did not meet the required CVI threshold across two Delphi rounds and were therefore excluded from the final framework. In Task 3, the newly added EPAs (18–21) were further evaluated for clarity and relevance in their Chinese-language versions, followed by another content validity assessment. All four EPAs achieved CVIs of 0.8 or higher, confirming their linguistic clarity and contextual appropriateness for implementation. As a result of this multi-step process, the final EPA framework for TCPC consisted of 21 validated EPAs.

The 21 EPAs and a corresponding core competency matrix are shown in Table 1. The 21-EPA program included the 17 items of the TSOP CBME framework, which covers general psychiatric practice, consideration of mental capacities, medical conditions and consultation, psychiatric public health, and resident faculty. In addition, newly added EPA items 18–21 concerned the need for more specialized skills and techniques, trainee’s mastery of long-term treatment goals, integration of legal and professional standards, and extension of innovative therapeutic techniques.

**Table 1:** List of the TCPC psychiatry 21 EPAs

No.	EPA description	Core competency domains					
		PC	MK	PBL	ICS	*P*	SBP
1	Assess a patient presenting with mental/behavioral symptoms & arrive at a psychiatric diagnosis.	+	+	+	+	+	
2	Screen patients for comorbid other medical conditions, identify medical problems that need attention.	+	+	+	+	+	+
3	Assess and manage risk of harm to self and others.	+	+	+	+	+	+
4	Formulate & implement an appropriate and comprehensive treatment plan for a patient with mental illness.	+	+	+	+	+	
5	Document and orally present a clinical encounter.	+	+	+	+	+	
6	Document & present all clinical data pertaining to a given psychiatric patient (history, examination, formulation, diagnoses, laboratory reports, treatment, progress, discharge plan etc.).	+	+	+	+	+	
7	Prepare succinct and informative diagnostic certificates.	+	+		+	+	
8	Form clinical questions and retrieve evidence to advance patient care.	+	+	+		+	
9	Give or receive a patient handover to transition care responsibility.	+	+	+	+	+	+
10	Obtain informed consent for tests and/or procedures.	+	+	+	+	+	+
11	Assess & manage mental/behavioral symptoms in a patient with other medical conditions, in collaboration with other medical specialists.	+	+	+	+	+	+
12	Perform all the expected tasks associated with the administration of electroconvulsive therapy (ECT).	+	+	+	+	+	+
13	Provide psychoeducation to a patient and their family and/or carers about a mental illness.	+	+	+	+	+	
14	Provide basic & accurate information about mental health & mental illness to the lay public, utilizing appropriate electronic & print media.		+		+	+	+
15	Conduct mental health screening in non-psychiatric settings.		+		+	+	+
16	Provide counselling about stress management, mental health promotion & prevention of mental illness.	+	+	+	+	+	+
17	Deliver didactic psychiatry lectures and conduct clinical training sessions for junior physicians, undergraduate medical & other medical personnel students.	+	+	+	+	+	+
18	Evaluate the appropriate psychological therapy intervention models for patients and can administer specific types of psychological therapy.	+	+	+	+	+	+
19	Understand and implement the mental health-related laws in clinical practice.	+	+	+	+	+	+
20	Assess and formulate rehabilitation plans and resource linkage following acute care.	+	+	+	+		+
21	Administer the repetitive transcranial magnetic stimulation (rTMS)	+	+	+	+	+	+

PC: patient care, MK: medical knowledge, PBL: practice-based learning and improvement, ICS: interpersonal and communication skills, P: medical professionalism, SBP: systems-based practice

### Discrepancies in perceptions of importance between trainees and teachers across the 21 EPAs

A comparison of the perceived importance of the 21 EPAs between attending psychiatrists (*n* = 27) and psychiatry residents (*n* = 16) is presented in e-Table 2. The results revealed statistically significant differences in the overall importance scores between the two groups. On average, attending psychiatrists consistently rated the EPAs as more important than residents did, with a total mean score of 9.04 (standard deviation [SD] = 0.94) compared to 8.23 (SD = 1.40) for residents (*p* = 0.049). Significant group differences emerged for several specific EPAs, including EPA 1 (*p* = 0.018), EPA 6 (*p* = 0.016), EPA 9 (*p* = 0.032), EPA 10 (*p* = 0.044), EPA 14 (*p* = 0.041), EPA 17 (*p* = 0.009), and EPA 20 (*p* = 0.018). These findings suggest that particular professional activities were valued differently by residents and their supervising faculty.

Further analysis using dichotomized data is shown in Table 2, where EPAs were classified based on whether respondents indicated full agreement regarding importance (Likert scale score = 10). Significant discrepancies in full agreement rates were observed for EPA 6 (documenting and presenting all clinical data related to a psychiatric patient), EPA 9 (giving or receiving patient handovers to transition care responsibilities), EPA 17 (delivering didactic psychiatry lectures and conducting clinical training sessions for junior physicians, undergraduate medical students, and other medical personnel students), and EPA 20 (assessing and formulating rehabilitation plans and facilitating resource linkage following acute care). In addition, a regression analysis was conducted to control for clinical working experience (measured by years of practice) to isolate the effect of trainee status on perceptions of importance. As shown in Table 3, resident status was associated with significantly lower importance ratings for EPA 1, EPA 6, EPA 10, EPA 17, and EPA 20. Notably, EPA 6, EPA 17, and EPA 20 consistently showed statistically significant group differences across e-Tables 2 and Table 2, and Table 3, suggesting that these EPAs represent key areas of perceptual divergence between trainees and faculty.

**Table 2:** Agreement of EPAs between attending psychiatrists and residents

EPAs	Total (*n* = 43)		Attending (*n* = 27)		Residence (*n* = 16)		*p*
	*N*	%	*N*	%	*N*	%	
EPA1							0.068
Not full agreement	11	25.58	4	14.81	7	43.75	
EPA2							0.280
Not full agreement	17	39.53	9	33.33	8	50.00	
EPA3							0.313
Not full agreement	12	27.91	6	22.22	6	37.50	
EPA4							0.141
Not full agreement	18	41.86	9	33.33	9	56.25	
EPA5							0.076
Not full agreement	22	51.16	11	40.74	11	68.75	
EPA6							0.018*
Not full agreement	25	58.14	12	44.44	13	81.25	
EPA7							0.137
Not full agreement	29	67.44	16	59.26	13	81.25	
EPA8							0.220
Not full agreement	19	44.19	10	37.04	9	56.25	
EPA9							0.032*
Not full agreement	26	60.47	13	48.15	13	81.25	
EPA10							0.088
Not full agreement	28	65.12	15	55.56	13	81.25	
EPA11							0.724
Not full agreement	20	46.51	12	44.44	8	50.00	
EPA12							0.324
Not full agreement	20	46.51	11	40.74	9	56.25	
EPA13							0.141
Not full agreement	18	41.86	9	33.33	9	56.25	
EPA14							0.416
Not full agreement	29	67.44	17	62.96	12	75.00	
EPA15							0.133
Not full agreement	26	60.47	14	51.85	12	75.00	
EPA16							0.295
Not full agreement	28	65.12	16	59.26	12	75.00	
EPA17							0.030*
Not full agreement	23	53.49	11	40.74	12	75.00	
EPA18							0.454
Not full agreement	21	48.84	12	44.44	9	56.25	
EPA19							0.405
Not full agreement	18	41.86	10	37.04	8	50.00	
EPA20							0.044*
Not full agreement	21	48.84	10	37.04	11	68.75	
EPA21							0.295
Not full agreement	28	65.12	16	59.26	12	75.00	

* *p* < 0.05

**Table 3:** Effect of being psychiatry resident to the importance of the EPAs

EPAs	β	SE	*p*
EPA1	-1.02	0.36	0.007**
EPA2	-1.16	0.41	0.008**
EPA3	-0.76	0.34	0.029*
EPA4	-0.83	0.42	0.055
EPA5	-1.05	0.52	0.049*
EPA6	-1.46	0.64	0.029*
EPA7	-1.14	0.97	0.245
EPA8	-1.32	0.59	0.031*
EPA9	-0.81	0.72	0.267
EPA10	-1.23	0.60	0.048*
EPA11	-0.68	0.52	0.202
EPA12	-0.86	0.51	0.101
EPA13	-1.41	0.60	0.024*
EPA14	-1.37	0.91	0.137
EPA15	-1.10	0.80	0.175
EPA16	-1.04	0.63	0.107
EPA17	-1.65	0.54	0.004**
EPA18	-0.94	0.49	0.065
EPA19	-0.88	0.46	0.063
EPA20	-1.30	0.46	0.007**
EPA21	-1.03	0.57	0.078
EPA (mean)	-1.10	0.43	0.015*

Working experience of the clinical practice (years) was included as a control variable, and the attending physician was used as the reference group

* *p* < 0.05; ** *p* < 0.01

### Factor analysis based on the importance of the EPAs

**Fig. 2:**
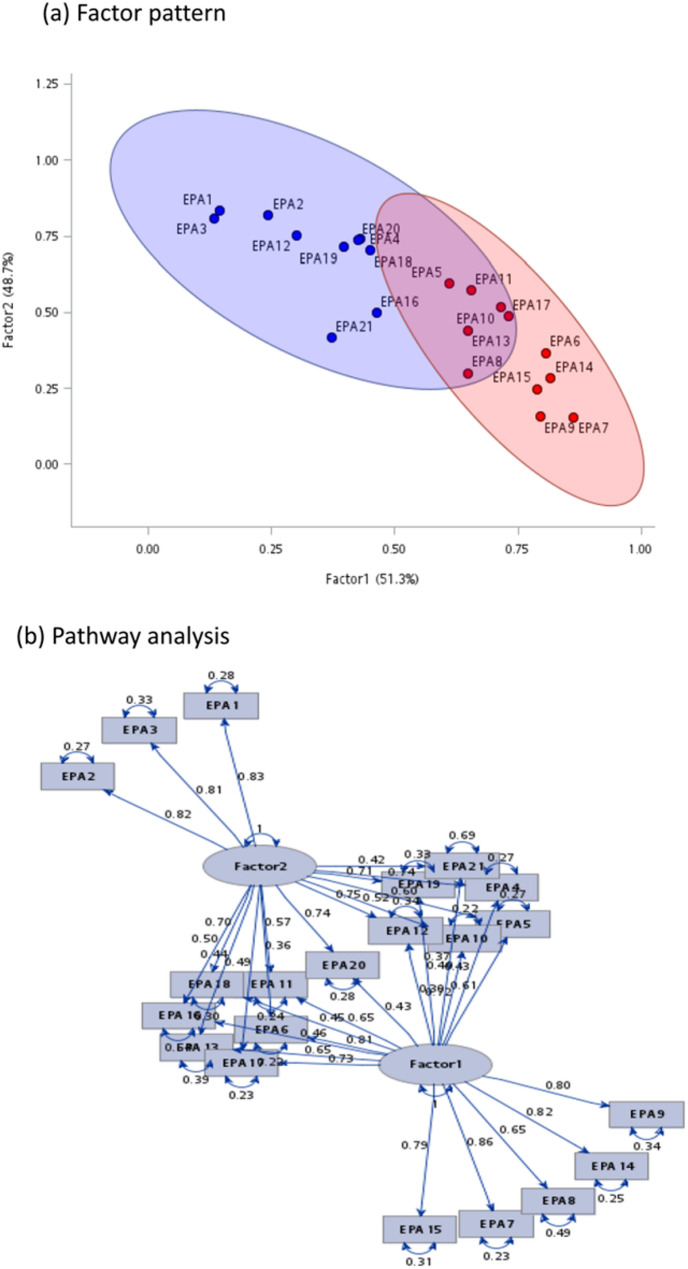
Factor analysis of the EPAs based on the importance

The factor analysis results based on the importance score identified two distinct factors, which explain a significant portion of the variance in the data. In the factor pattern (Fig. 2a), the scatter plot visually demonstrates the distribution of EPAs across the two factors. Factor 1 explains 51.3% of the variance, while factor 2 accounts for an additional 48.7%. The pathway analysis further illustrates the relationships between individual EPAs and their respective factors (Fig. 2b). EPAs in factor 1 (red cluster) exhibit strong loadings and include EPA 7, 8, 9, 14, and 15, which emphasize the role of communication and information management in psychiatric practice. EPAs in factor 2 (blue cluster), such as EPA 1, 2, and 3, reflect the importance of core clinical reasoning and decision-making processes. EPAs with cross-factor pathways indicate some degree of overlap between the two domains, but the factors remain largely distinct. The suitability of the data for factor analysis was supported by a Kaiser–Meyer–Olkin (KMO) measure of sampling adequacy of 0.73 overall, with all individual EPAs yielding KMO values greater than 0.5.

## Discussion

### The meaningful process of tailoring EPAs to institutional contexts

The development of EPAs involves reflective approaches to the daily work of professionals; therefore, the creation of context-specific EPAs at individual hospitals remains valuable [12, 13]. With the establishment of a national EPA framework by the TSOP, this is a timely opportunity for individual hospitals to evaluate the framework’s applicability and to consider developing specialized EPAs that reflect their unique clinical settings and educational strengths. Items 18 to 21 differ from the previous 17 items in terms of depth, focus, and the application of specialized skills. The first 17 items primarily establish foundational clinical skills such as history taking, diagnosis, risk management, and psychoeducation, while items 18 to 21 introduce more specialized and technical abilities. For example, EPA 18 involves evaluating psychological therapy models and administering specific therapies, which require a deeper understanding of therapeutic approaches and the ability to personalize care. EPA 19 emphasizes the integration of legal and ethical considerations into psychiatric practice, highlighting the psychiatrist’s broader societal responsibilities. EPA 20 focuses on formulating rehabilitation plans, linking patients to essential resources, and addressing long-term care needs that go beyond immediate treatment. EPA 21 highlights the administration of repetitive transcranial magnetic stimulation (rTMS) and demonstrates the importance of mastering innovative psychiatric interventions. Together, these advanced EPAs not only broaden the scope of psychiatric training but also prepare residents to become specialized, forward-thinking practitioners equipped to meet evolving clinical and societal needs.

Comparisons between the TSOP framework with international literature are shown in supplementary e-Table 3 (milestones) and e-Table 4 (EPAs). The TCPC EPAs incorporate several distinctive components not explicitly outlined in the AADPRT Psychiatry Task Force framework. Conversely, they also omit certain EPAs that are highlighted in the AADPRT version. For example, TCPC EPA 18 (evaluating psychological therapy models and administering specific types) is partially covered in AADPRT EPAs 9, 11, and 12 to emphasize evaluating and implementing specific psychological therapy models, as well as detailing therapeutic interventions. TCPC EPA 19 (implementing mental health-related laws) is not explicitly covered by the AADPRT and refers to the relevance in local healthcare systems. TCPC EPA 20 (assessing and formulating rehabilitation plans and resource linkage) aligns with the concept of long-term patient care in the concept of AADPRT EPA 1. TCPC EPA 21 is related to administering rTMS, and although not directly covered by AADPRT, it is one of the key development focuses and is one of the hospital’s teaching features. The AADPRT EPAs also include leadership in inter-professional healthcare teams and the application of quality-improvement methodologies in clinical practice. These elements were considered essential and discussed during the development process. However, consensus was not reached on these two EPAs, as the CBME team viewed them as competencies more appropriate for experienced attending physicians and noted the challenges associated with evaluating them during residency training.

### Perceptions of the importance of the 21 EPAs between trainees and teachers

These findings indicate that teachers are more aware of the importance of EPAs for trainees and also reflect the gap between teachers’ and trainee’s expectations about advanced experience. EPA 6, EPA 17, and EPA 20 were especially noted through kinds of statistical methods. Perceptions of importance differ between trainees and supervisors regarding EPA 6 (documenting and presenting all clinical data related to a given psychiatric patient), EPA 17 (delivering didactic psychiatry lectures and conducting clinical training for junior physicians, medical students, and other healthcare trainees), and EPA 20 (assessing and formulating rehabilitation plans and establishing resource linkages following acute care), may reflect differing perspectives on future professional roles. Trainees may consider these activities—such as comprehensive documentation, teaching, and rehabilitation planning—as peripheral to their anticipated career paths. For instance, EPA 6 may be undervalued because residents rarely directly encounter legal or medical disputes arising from documentation errors during training. Similarly, EPA 17 might be seen as less relevant by those who do not plan to work in teaching hospitals for their future career, leading them to underestimate the value of teaching medical students. EPA 20 may be underappreciated due to limited opportunities for junior trainees to follow patients longitudinally, making it difficult for them to grasp the importance of long-term care planning. From this point of view, this misalignment may hinder trainee engagement and preparation. It is therefore important to better align expectations and to contextualize the relevance of each EPA within diverse psychiatric career trajectories, in order to address the gaps identified by trainees [14, 15].

### Factor analysis of the importance of the EPAs

Based on the importance ratings, EPAs were clustered to two main factors. Factor 2 (blue cluster) emphasizes the ability to diagnose patients, assess risks, and screen for comorbid conditions, all of which rely heavily on a resident’s internal knowledge, judgment, and clinical evaluation skills. Factor 2 is related to knowledge and clinical reasoning competence and focuses on the core clinical skills required for psychiatric residents. This factor represents the foundational cognitive processes that enable psychiatrists to act as effective clinical decision-makers. Factor 1 (red cluster) is related to EPAs for communication and information delivery skills and highlights the outward-facing abilities of psychiatric residents to convey their knowledge and clinical judgments effectively. It encompasses skills such as documenting clinical records, applying knowledge in practice, educating patients, collaborating across professional teams, and promoting mental health awareness. This factor reflects the capacity to externalize professional expertise through communication, documentation, and educational efforts to ensure effective interaction with patients, families, and colleagues.

Sharing the results of the factor analysis with both faculty and trainees may enhance their engagement in competency-based training. For example, the factor loading characteristics of EPAs prompt further consideration of whether they reflect individual learning styles. Individual learner characteristics—such as their tendencies toward interactive versus reflective learning, and their preferred modes of knowledge acquisition—may correspond to their rating categorization of EPAs. Learners who are more inclined toward interactive, communication-based learning may naturally engage more effectively and rating importantly with Factor 1 related EPAs, which emphasize interpersonal communication, education, and collaborative practice. In contrast, those who prefer reflective or solitary learning may align and rate more closely with Factor 2 EPAs, which involve internal clinical reasoning, diagnostic assessment, and analytical decision-making. Similarly, learners who favor text-based study methods may feel more comfortable with the cognitive demands of Factor 2, while those who thrive in experiential, hands-on environments may relate more intuitively to the practice-oriented aspects of Factor 1. By understanding these learner characteristics and learning styles through their rating of importance among EPAs, we may be able to enhance the educational development of trainees, to design teaching strategies that cater to various traits and learning styles, to better suit their individual needs and support diverse developmental goals [16].

### Implications and future works

Alongside the first step of the TSOP CBME development, in which CBME-developed international version turn into CBME-developing country’s national guide, this study further enables the framework to be better aligned with the localized context. By adapting global and national standards, the framework becomes more helpful for local teachers and trainees [16–18]. Although this study did not use the standard EQual criteria to validate EPAs [19], we explored perceived importance ratings and observed that certain EPAs may be underestimated by trainees. Through factor analysis, we conceptualized the EPAs into two main dimensions: one related to internal clinical knowledge and reasoning, and the other to external communication and collaboration.

Currently, in our institution, residents typically work under indirect supervision and are evaluated based on the six core competencies. After completing four years of required clinical training and participating in core reading sessions, they are eligible to take the written and interview examinations administered by the RRC. Upon passing these examinations, they are considered to have obtained board certification. Therefore, as we gradually incorporate the principles of CBME into our current training and assessment system, it is important for both faculty and trainees to understand the origins and development of local EPAs. We believe this approach can positively influence both teaching practices and learning motivation [20]. We are interested about whether a growth mindset in the context of CBME or the sense of importance of the EPA tasks may impact the outcomes of learning or patient care in clinical education [21, 22]. We plan to follow up on the teaching and learning outcomes in a context where teachers and trainees are already aware of the factors related to EPA importance. The next step for TSOP psychiatry CBME development is to engage in pilot testing across approximately 10 institutions. The experiences from this study will serve as a valuable reference to guide the selection of EPAs for pilot testing and to facilitate feedback collection from learners and supervisors.

When finalizing the three steps for the TSOP CBME implementation in psychiatry residency training, the levels of supervision will provide a structured way to assess the degree of independence a learner can be entrusted with while performing a professional task. The model outlines five levels of supervision based on Ten Cate’s framework (2005) [23], which are critical for making entrustment decisions. The evaluation of trainees’ competencies will be integrated into current four-year training program at qualified training institutes. The formal decisions regarding whether a trainee can independently perform a professional activity are typically made by a Competence Committee (CCC) at the qualified training institutes. These decisions should be based on multiple data points, including direct observations, EPA assessments, and feedback from various supervisors, ensuring a comprehensive and objective evaluation of the learner’s readiness.

## Conclusions

In this study, we developed the TCPC EPAs based on the TSOP framework, encompassing both foundational clinical skills and broader competencies in psychiatric practice. The framework aimed to support residents’ progression toward advanced expertise, particularly in psychotherapy, long-term rehabilitation, legal and ethical practice, and emerging psychiatric technologies. This experience of “localizing” EPAs demonstrates how adapting global or national standards to institutional contexts can enhance their relevance and usefulness for local training institutions. Additionally, we identified a misalignment in perceptions of EPA importance between trainees and teachers, which may impede trainee engagement and preparedness. Factor analysis of the importance ratings revealed two core clusters of EPAs: one reflecting internal knowledge and clinical reasoning, and the other emphasizing external communication and information delivery. By increasing awareness and understanding of these underlying dimensions among both trainees and instructors, and by applying the EPAs in a more personalized manner, we anticipate that the effectiveness of psychiatric education and training can be significantly improved.

## Electronic supplementary material

Below is the link to the electronic supplementary material.

Supplementary Material 1

## Data Availability

The data that support the findings of this study are available on request from the corresponding author.
