# Supplementary material for: Importance-based approach to entrustable professional activities for psychiatric residency training
Source: BMC Med Educ. 2025 Jul 17;25:1068. doi: 10.1186/s12909-025-07655-0 (PMC12273430; doi:10.1186/s12909-025-07655-0)
Supplement: Supplementary file 1 — Supplementary Material 1 [file 12909_2025_7655_MOESM1_ESM.docx]

**Supplement**

**List of e-Tables**

**e-Table 1.** The percent of the mode (Mo) and the quartile deviation (QD) for the suitability assessed by the TCPC CBME team

**e-Table 2.** EPA importance score between attending psychiatrists and residents

**e-Table 3.** The comparisons of milestones between TSOP framework with ACGME 2020 revision

**e-Table 4.** The comparisons of EPAs between TSOP framework with AADPRT EPA Psychiatry Task Force

**e-Table 1.** The percent of the mode (Mo) and the quartile deviation (QD) for the suitability assessed by the TCPC CBME team

| EPAs | | Task 1 | | | | Task 2 | | | Task 3 | | |
| --- | --- | --- | --- | --- | --- | --- | --- | --- | --- | --- | --- |
|  | Round one  Mo (%) / QD | | Round two  Mo (%) / QD | final  CVI | Round one  Mo (%) / QD | Round two  Mo (%) / QD | final  CVI | Round one  Mo (%) / QD | | Round two  Mo (%) / QD | final  CVI |
| 1 | 5 (87.5) / 0.5 | | - | 1 |  |  |  |  | |  |  |
| 2 | 5 (75) / 0.5 | | - | 1 |  |  |  |  | |  |  |
| 3 | 5 (100) / 0 | | - | 1 |  |  |  |  | |  |  |
| 4 | 5 (50) / 0.5 | | - | 1 |  |  |  |  | |  |  |
| 5 | 4 (62.5) / 0.5 | | - | 1 |  |  |  |  | |  |  |
| 6 | 4 (75) / 0.5 | |  | 0.875 |  |  |  |  | |  |  |
| 7 | 3 (50) / 1.5 | | 4 (87.5)* / 0.5 | 0.875 |  |  |  |  | |  |  |
| 8 | 4 (50) / 1.5 | | - | 0.875 |  |  |  |  | |  |  |
| 9 | 4 (62.5) / 2 | | - | 1 |  |  |  |  | |  |  |
| 10 | 4 (87.5) / 0.5 | | - | 0.875 |  |  |  |  | |  |  |
| 11 | 5 (50) / 0.5 | | - | 1 |  |  |  |  | |  |  |
| 12 | 5 (50) / 0.5 | | - | 1 |  |  |  |  | |  |  |
| 13 | 5 (62.5) / 0.5 | | - | 1 |  |  |  |  | |  |  |
| 14 | 3 (62.5) / 1.5 | | 4 (50)* / 1.5 | 0.875 |  |  |  |  | |  |  |
| 15 | 4 (37.5) / 2 | | 4 (62.5)* / 2 | 0.875 |  |  |  |  | |  |  |
| 16 | 4 (50) / 1.5 | | - | 0.875 |  |  |  |  | |  |  |
| 17 | 5 (50) / 1.5 | | - | 1 |  |  |  |  | |  |  |
| 18 | - | | - |  | 5 (50) / 0.5 | - | 1 | 5 (62.5) / 0.5 | | 5 (75)* / 0.5 | 1 |
| 19 | - | | - |  | 5 (62.5) / 0.5 | - | 1 | 5 (62.5) / 0.5 | | 5 (62.5) / 0.5 | 1 |
| 20 | - | | - |  | 4 (62.5) / 0.5 | - | 1 | 4 (62.5) / 0.5 | | 5 (50)* / 0.5 | 1 |
| 21 | - | | - |  | 4 (62.5) / 1.5 | - | 0.875 | 4 (50) / 1 | | 4 (50) / 1 | 0.875 |
| Excluded EPA a |  | |  |  | 3 (75) / 0.5 | 3 (75) / 0.5 | 0.25 |  | |  |  |
| Excluded EPA b |  | |  |  | 3 (62.5) / 0.5 | 3 (62.5) / 0.5 | 0.125 |  | |  |  |

Excluded EPA a : leadership in inter-professional healthcare teams

Excluded EPA b : application of quality-improvement methodologies in clinical practice.

*Percent of the mode changed in round two

**e-Table 2.** EPA importance score between attending psychiatrists and residents

| EPAs | | Total (n=43) | | | Attending (n=27) | | | | Residence (n=16) | | | | | p |
| --- | --- | --- | --- | --- | --- | --- | --- | --- | --- | --- | --- | --- | --- | --- |
|  |  | Mean | | SD | Mean | | SD | | Mean | | SD | | |  |
| EPA1 | 9.54 | | 1.01 | | | 9.85 | | 0.36 | | 9.00 | | 1.46 | 0.018* | |
| EPA2 | 9.30 | | 1.15 | | | 9.56 | | 0.75 | | 8.88 | | 1.54 | 0.147 | |
| EPA3 | 9.58 | | 0.91 | | | 9.74 | | 0.53 | | 9.31 | | 1.30 | 0.249 | |
| EPA4 | 9.26 | | 1.12 | | | 9.41 | | 0.97 | | 9.00 | | 1.32 | 0.214 | |
| EPA5 | 8.91 | | 1.38 | | | 9.19 | | 1.15 | | 8.44 | | 1.63 | 0.093 | |
| EPA6 | 8.37 | | 1.75 | | | 8.85 | | 1.56 | | 7.56 | | 1.79 | 0.016* | |
| EPA7 | 7.40 | | 2.51 | | | 7.78 | | 2.34 | | 6.75 | | 2.72 | 0.213 | |
| EPA8 | 8.84 | | 1.59 | | | 9.22 | | 1.22 | | 8.19 | | 1.94 | 0.081 | |
| EPA9 | 8.35 | | 1.88 | | | 8.70 | | 1.94 | | 7.75 | | 1.65 | 0.032* | |
| EPA10 | 8.42 | | 1.62 | | | 8.82 | | 1.33 | | 7.75 | | 1.88 | 0.044* | |
| EPA11 | 8.98 | | 1.35 | | | 9.11 | | 1.19 | | 8.75 | | 1.61 | 0.583 | |
| EPA12 | 8.98 | | 1.35 | | | 9.15 | | 1.20 | | 8.69 | | 1.58 | 0.337 | |
| EPA13 | 8.98 | | 1.63 | | | 9.37 | | 1.12 | | 8.31 | | 2.12 | 0.074 | |
| EPA14 | 7.65 | | 2.44 | | | 8.26 | | 2.03 | | 6.63 | | 2.78 | 0.041* | |
| EPA15 | 8.26 | | 2.07 | | | 8.52 | | 2.24 | | 7.81 | | 1.72 | 0.077 | |
| EPA16 | 8.28 | | 1.65 | | | 8.48 | | 1.60 | | 7.94 | | 1.73 | 0.318 | |
| EPA17 | 8.70 | | 1.55 | | | 9.22 | | 1.09 | | 7.81 | | 1.83 | 0.009** | |
| EPA18 | 9.00 | | 1.31 | | | 9.22 | | 1.12 | | 8.63 | | 1.54 | 0.211 | |
| EPA19 | 9.26 | | 1.22 | | | 9.44 | | 0.85 | | 8.94 | | 1.65 | 0.395 | |
| EPA20 | 9.05 | | 1.29 | | | 9.44 | | 0.85 | | 8.38 | | 1.63 | 0.018* | |
| EPA21 | 8.40 | | 1.58 | | | 8.44 | | 1.67 | | 8.31 | | 1.45 | 0.650 | |
| Total | 8.74 | | 1.18 | | | 9.04 | | 0.94 | | 8.23 | | 1.40 | 0.049* | |

* *p* < 0.05; ** *p* < 0.01

**e-Table 3.** The comparisons of milestones between TSOP framework with ACGME 2020 revision

| ACGME 2020 revision ^a^ | TSOP framework / Chinese version ^b^ |
| --- | --- |
| Patient Care (PC) | |
| PC 1: Psychiatric Evaluation | PC 1. Psychiatric Evaluation / 精神評估 |
| PC 2: Psychiatric Formulation and Differential Diagnosis | PC 2. Psychiatric Formulation and Differential Diagnosis / 精神醫學的架構和鑑別診斷 |
| PC 3: Treatment Planning and Management | PC 3. Treatment Planning and Management / 治療計劃和處置 |
| PC 4: Psychotherapy | PC 4. Psychotherapy/ 心理治療 |
| PC5: Somatic Therapies (including Psychopharmacology and Neurostimulation Therapies) | PC 5. Somatic Therapies (including Psychopharmacology and Neurostimulation Therapies) / 身心治療(包括精神藥理學，電痙攣治療和新興神經調節療法) |
| PC 6: Clinical Consultation | - |
| Medical Knowledge (MK) | |
| MK 1: Development through the Life Cycle (including the Impact of Psychopathology on the Trajectory of Development and Development on the Expression of Psychopathology) | MK 1. Development through the Life Cycle (including the Impact of Psychopathology on the Trajectory of Development and Development on the Expression of Psychopathology) / 生命週期的發展（包括精神病理學對發展的影響，以及發展對精神病理學表現的影響） |
| MK 2: Psychopathology (includes Knowledge of Diagnostic Criteria, Epidemiology, Pathophysiology, Course of Illness, Comorbidities, and Differential Diagnosis of Psychiatric Disorders, including Substance Use Disorders and Presentation of Psychiatric Disorders across the Life Cycle and in Diverse Patient Populations) | MK 2. : Psychopathology (includes Knowledge of Diagnostic Criteria, Epidemiology, Pathophysiology, Course of Illness, Comorbidities, and Differential Diagnosis of Psychiatric Disorders, including Substance Use Disorders and Presentation of Psychiatric Disorders across the Life Cycle and in Diverse Patient Populations, such as different cultures, families, genders, sexual orientations, races, etc.) / 精神病理學 (包括診斷標準，流行病學，病理生理學，病程，合併症和精神疾病的鑑別診斷知識，包括物質使用疾患和涵蓋整個生命週期及不同病人族群，例如，不同文化， 家庭，性別，性取向，種族等） |
| MK 3: Clinical Neuroscience (includes Knowledge of Neurology, Neuropsychiatry, Neurodiagnostic Testing, and Relevant Neuroscience and their Application in Clinical Setting | MK 3. Clinical Neuroscience (includes Knowledge of Neurology, Neuropsychiatry, Neurodiagnostic Testing, and Relevant Neuroscience and their Application in Clinical Settings) / 臨床神經科學 (包括神經學，神經精神病學，神經診斷測試和相關神經科學知識及其在臨床環境中的應用) |
| MK 4: Psychotherapy | MK 4. Psychotherapy / 心理治療 |
| - | MK 5. Somatic Therapies: Knowledge of somatic treatments, including psychopharmacology, electroconvulsive therapy, and emerging Somatic therapies such as transcranial magnetic stimulation and vagus nerve stimulation / 身體治療：身體治療的知識，包括精神藥理學、電痙攣治療和新興的身體療法，如經顱磁刺激術和迷走神經刺激術 |
| - | MK 6. Psychiatric practice / 精神醫學實踐 |
| Systems-Based Practice (SBP) | |
| SBP 1: Patient Safety and Quality Improvement | SBP 1. Patient Safety and Quality Improvement of Health Care Team /病人安全和醫療照顧團隊 |
| SBP 2: System Navigation for Patient-Centered Care | SBP 2. Resource management (which may include diagnostics, medications, level of care, other treatment providers, and access to community support) / 資源管理（可能包括診斷、藥物、照護水平、其他治療提供者、社區援助的取得） |
| SBP 3: Physician Role in Health Care Systems | SBP 3. Psychiatrist in Community care / 社區照顧 |
| Practice-Based Learning and Improvement (PBLI) | |
| PBLI 1: Evidence-Based and Informed Practice | PBLI 1. Develop and implement lifelong learning through continuous self-assessment, including evidence-based research evaluation / 透過持續的自我評量(包括嚴謹而具實證等級的研究評估)來發展與執行終生學習 |
| PBLI 2: Reflective Practice and Commitment to Personal Growth | PBLI 2. Plan and implement a formal quality improvement program based on a comprehensive framework / 基於完整架構規劃出正規之品質促進計畫 |
| - | PBLI 3. Medical teaching /教學及醫學教育 |
| Professionalism (PROF) | |
| PROF 1: Professional Behavior and Ethical Principles | PROF 1. Empathy, integrity, and respect for others, with sensitivity to diverse patient populations and adherence to ethical principles / 同理，誠信，尊重他人，對不同患者群體具敏感度 且遵守道德原則 |
| PROF 2: Accountability/Conscientiousness | PROF 2. Responsibility to oneself, patients, peers, and the profession / 對自己，患者，同儕和專業的責任 |
| PROF 3: Well-Being | - |
| Interpersonal and Communication Skills (ICS) | |
| ICS 1: Patient- and Family-Centered Communication | ICS1. Relationship development and conflict management with patients, cargivers, colleagues, and healthcare team members / 與患者，家屬，同事和醫療團隊成員的關係發展和衝突管理 |
| ICS 2: Interprofessional and Team Communication | ICS2. Information sharing and record keeping / 訊息共享和記錄保存 |
| ICS 3: Communication within Health Care Systems | - |

^a^ cited from 2020 Accreditation Council for Graduate Medical Education (ACGME)

^b^ TSOP framework (Prof. Cheng-Sheng Chen et. al.)

**e-Table 4.** The comparisons of EPAs between TSOP framework with AADPRT EPA Psychiatry Task Force

| American Association of Directors of Psychiatry Residency Training (AADPRT) EPA Psychiatry Task Force ^a^ | | TSOP framework ^b^ | |
| --- | --- | --- | --- |
| 1 | Manage psychiatric patients longitudinally | - | - |
| 2 | Manage psychiatric emergencies | 3 | Assess and manage risk of harm to self and others |
| 3 | Conduct psychiatric diagnostic evaluations | 1 | Assess a patient presenting with mental/behavioral symptoms & arrive at a psychiatric diagnosis. |
| 4 | Manage patient’s psychiatric conditions with medications | 4 | Formulate & implement an appropriate and comprehensive treatment plan for a patient with mental illness. |
| 5 | Manage involuntary commitment and treatment | - | - |
| 6 | Assess and manage decision-making capacity | - | - |
| 7 | Manage transitions in care | 9 | Give or receive a patient handover to transition care responsibility. |
| 8 | Provide psychiatric consultation to other clinicians or services | 11 | Assess & manage mental/behavioral symptoms in a patient with other medical conditions, in collaboration with other medical specialists. |
| 9 | Provide supportive psychotherapy | - | - |
| 10 | Lead an interprofessional health care team | - | - |
| 11 | Provide cognitive behavioral therapy | - | - |
| 12 | Provide psychodynamic psychotherapy | - | - |
| 13 | Apply quality improvement methodologies to one’s patient panel or clinical service | - | - |
| - | - | 2 | Screen patients for comorbid other medical conditions, identify medical problems that need attention. |
| - | - | 5 | Document and orally present a clinical encounter. |
| - | - | 6 | Document & present all clinical data pertaining to a given psychiatric patient (history, examination, formulation, diagnoses, laboratory reports, treatment, progress, discharge plan etc.). |
| - | - | 7 | Prepare succinct and informative diagnostic certiﬁcates. |
| - | - | 8 | Form clinical questions and retrieve evidence to advance patient care. |
| - | - | 10 | Obtain informed consent for tests and/or procedures. |
| - | - | 12 | Perform all the expected tasks associated with the administration of electroconvulsive therapy (ECT). |
| - | - | 13 | Provide psychoeducation to a patient and their family and/or carers about a mental illness. |
| - | - | 14 | Provide basic & accurate information about mental health & mental illness to the lay public, utilizing appropriate electronic & print media. |
| - | - | 15 | Conduct mental health screening in non-psychiatric settings. |
| - | - | 16 | Provide counselling about stress management, mental health promotion & prevention of mental illness. |
| - | - | 17 | Deliver didactic psychiatry lectures and conduct clinical training sessions for junior physicians, undergraduate medical & other medical personnel students. |

^a^ cited from Academic Medicine, Vol 93, No 7/ July 2018

^b^ TSOP framework (Prof. Cheng-Sheng Chen et. al.)
